# Correction: Activation of Notch Signaling Is Required for Cholangiocarcinoma Progression and Is Enhanced by Inactivation of p53 *In Vivo*

**DOI:** 10.1371/journal.pone.0206953

**Published:** 2018-11-01

**Authors:** Mona El Khatib, Przemyslaw Bozko, Vindhya Palagani, Nisar P. Malek, Ludwig Wilkens, Ruben R. Plentz

## Notice of republication

This article [1] was republished on October 22, 2018 to address concerns raised post-publication regarding the Figs 3, 4, and 5.

- The image presented for the DMSO/SZ1 experiment in Fig 3A was duplicated from a previous publication (Fig 5A in [2]).
- In Fig 4, duplicate images were shown for SZ1 treated with DMSO and 5μM GSI IX, and the wrong image was shown for the 20μM panel.
- In Fig 5, duplicate images were presented in the “Snail” and “E cadherin” panels of Fig 5A and Fig 5B, and the molecular weight labels for these panels in Fig 5B were incorrect.

The University of Tuebingen investigated these issues and did not find evidence of misconduct.

The authors apologize for the errors in the original publication [1] and have provided corrected versions of the above figures which are included in the republished version. In addition, the raw blot images for Fig 5 are now provided in Supporting Information (S1 File).

Please download this article again to view the correct version.

## Supporting information

S1 File Raw blots for Fig 5. (PPT)

## References

[1] El Khatib M., Bozko P., Palagani V., Malek N. P., Wilkens L., Plentz R. R. (2013) Activation of Notch Signaling Is Required for Cholangiocarcinoma Progression and Is Enhanced by Inactivation of p53 *In Vivo*. PLoS ONE 8(10): e77433 10.1371/journal.pone.0077433 24204826 PMC3813685

[2] El Khatib M., Kalnytska A., Palagani V., Kossatz U., Manns M. P., Malek N. P., et. al (2013) Inhibition of hedgehog signaling attenuates carcinogenesis *in vitro* and increases necrosis of cholangiocellular carcinoma. Hepatology, 57: 1035–1045. 23172661 10.1002/hep.26147

