# Supplementary material for: Correction: Activation of Notch Signaling Is Required for Cholangiocarcinoma Progression and Is Enhanced by Inactivation of p53 In Vivo
Source: PLoS One. 2018 Nov 1;13(11):e0206953. doi: 10.1371/journal.pone.0206953 (PMC6211738; doi:10.1371/journal.pone.0206953)
Supplement: S1 File — (PPT) [file pone.0206953.s001.ppt]

## Slide 1
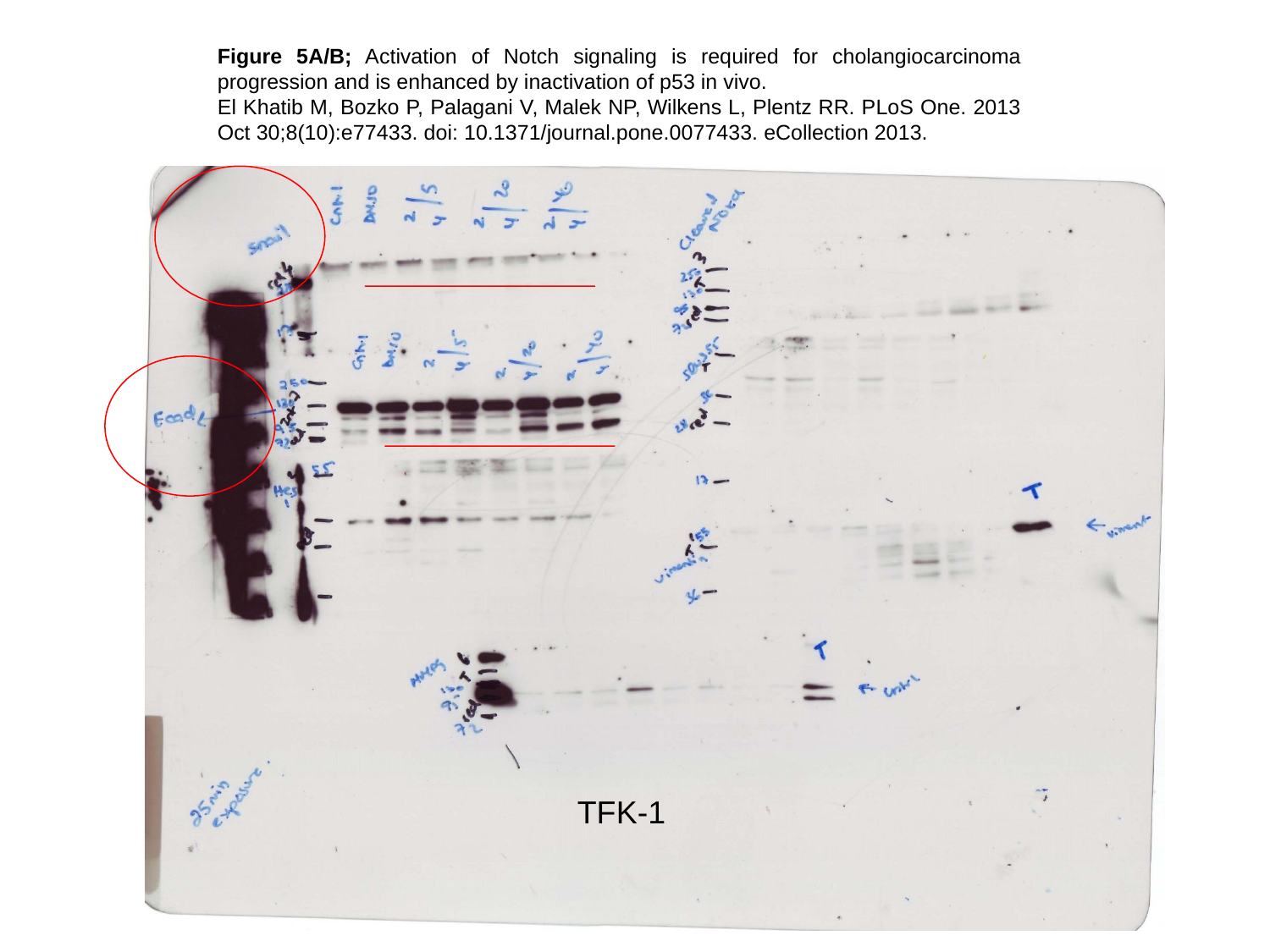

Figure 5A/B; Activation of Notch signaling is required for cholangiocarcinoma progression and is enhanced by inactivation of p53 in vivo.
El Khatib M, Bozko P, Palagani V, Malek NP, Wilkens L, Plentz RR. PLoS One. 2013 Oct 30;8(10):e77433. doi: 10.1371/journal.pone.0077433. eCollection 2013.
TFK-1

## Slide 2
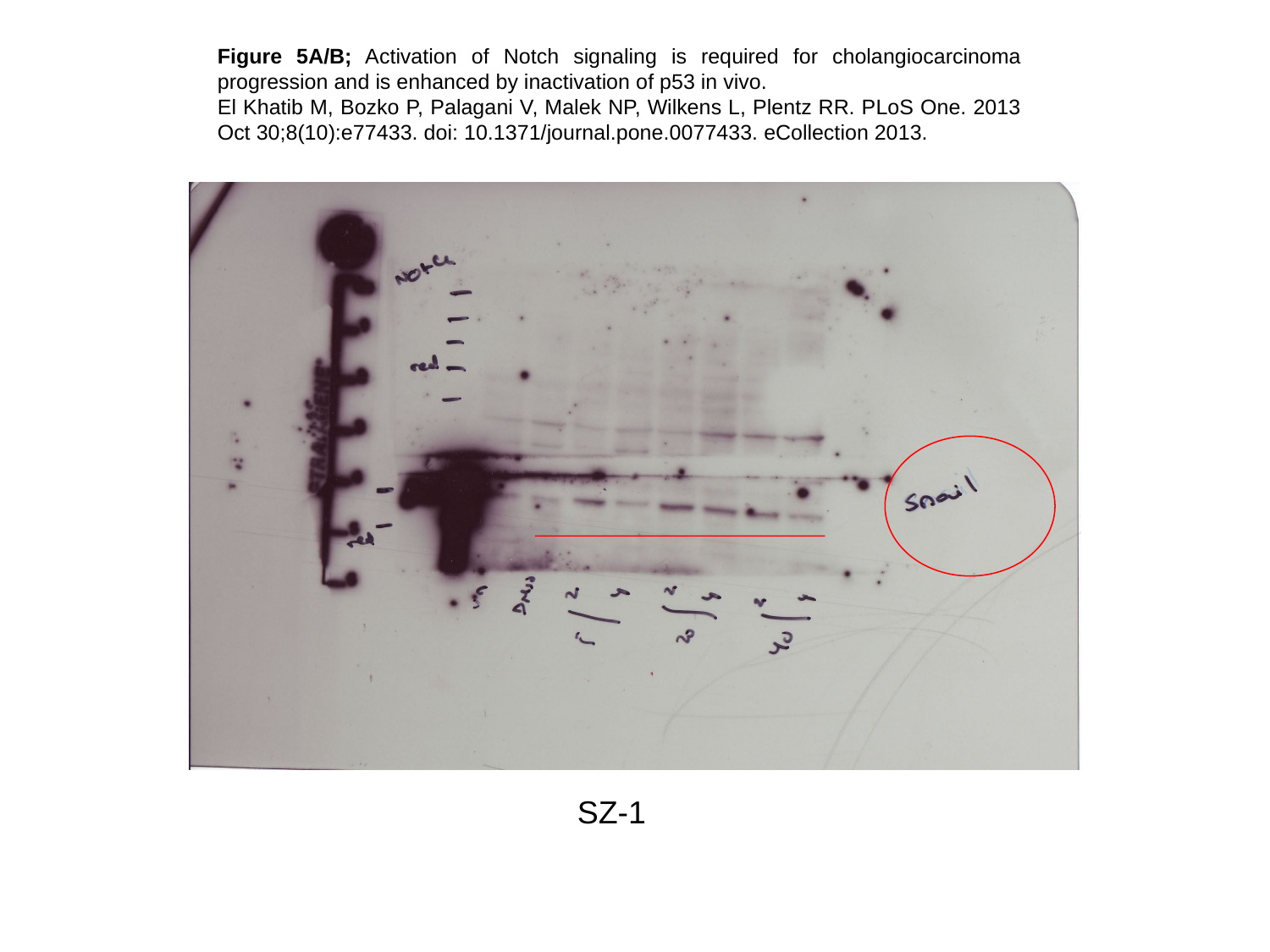

Figure 5A/B; Activation of Notch signaling is required for cholangiocarcinoma progression and is enhanced by inactivation of p53 in vivo.
El Khatib M, Bozko P, Palagani V, Malek NP, Wilkens L, Plentz RR. PLoS One. 2013 Oct 30;8(10):e77433. doi: 10.1371/journal.pone.0077433. eCollection 2013.
SZ-1

## Slide 3
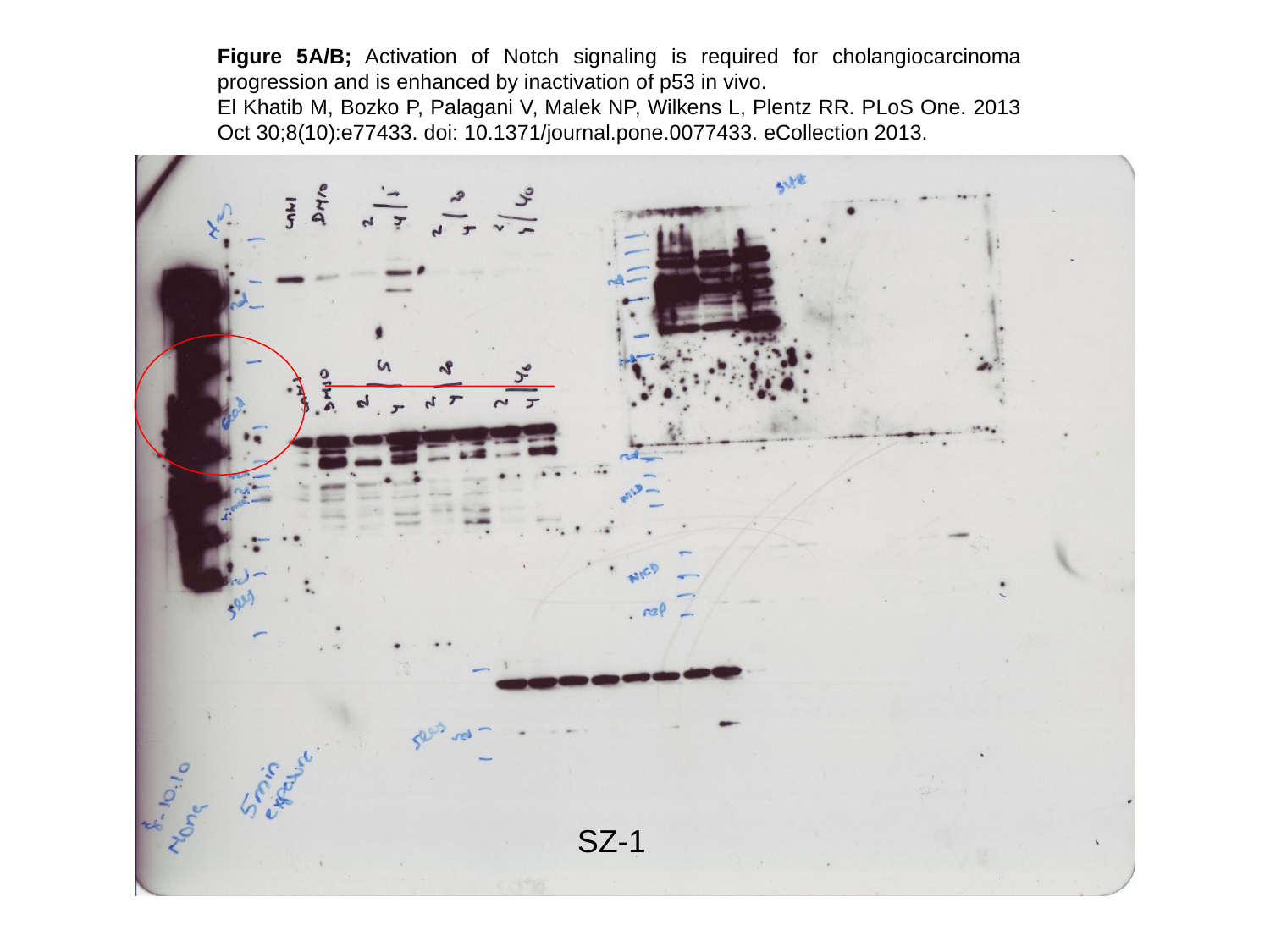

Figure 5A/B; Activation of Notch signaling is required for cholangiocarcinoma progression and is enhanced by inactivation of p53 in vivo.
El Khatib M, Bozko P, Palagani V, Malek NP, Wilkens L, Plentz RR. PLoS One. 2013 Oct 30;8(10):e77433. doi: 10.1371/journal.pone.0077433. eCollection 2013.
SZ-1
